# Medicine in the Mother Tongue: Navigating Linguistic Realities in Syrian Education

**DOI:** 10.1055/s-0045-1811692

**Published:** 2025-09-26

**Authors:** Hany Habib, M-Nasan Abdul Baki

**Affiliations:** 1 Department of Internal Medicine, Allegheny Health Network, Pittsburgh, Pennsylvania, United States; 2 Department of Pathology and Laboratory Medicine, Allegheny General Hospital, Pittsburgh, Pennsylvania, United States

For over a century, Arabic has served as the primary language of instruction in Syrian medical universities. This policy, emphasizing national identity and educational accessibility, aligns with the global practice of utilizing native languages to enhance student comprehension and engagement. Countries known for their robust medical education systems, including Japan, Germany, and China, similarly prioritize their native languages to foster deeper learning and contextual understanding. 1

However, the continued reliance on Arabic in Syrian medical education faces several notable challenges. Institutional constraints, limited availability of updated Arabic language medical textbooks, and the persistence of outdated terminology significantly contribute to educational shortcomings. Consequently, students frequently turn to English language resources to access the most current medical knowledge, a practice that can present practical difficulties and academic inefficiencies.

Emerging research indicates that transitioning entirely to English instruction may not be a straightforward remedy. Studies demonstrate that medical students educated in a nonnative language often experience lower comprehension levels, decreased academic performance, increased anxiety, and higher dropout rates. Conversely, education in one's native language can enhance conceptual understanding and reduce cognitive burden, especially during foundational clinical training. 2

Beyond academia, language proficiency holds critical implications for clinical practice. Competence in a patient's native language correlates positively with improved clinical communication, enhanced patient satisfaction, and better healthcare outcomes, principles fundamental to equitable healthcare delivery. 3 Thus, preserving Arabic fluency among medical trainees is beneficial not only educationally but also in clinical interactions within the broader Arabic-speaking community.

Nevertheless, Syrian medical students continue to face systemic limitations. The scarcity of updated Arabic language medical resources hampers their ability to stay abreast of international advancements. Bridging this educational gap necessitates targeted investments in translation efforts, terminology standardization, and the development of accessible Arabic language medical literature. Until such resources are adequately provided, students must navigate a dual linguistic environment, studying primarily in Arabic while frequently consulting English language references.

The Syrian experience highlights a vital global challenge in medical education: reconciling local accessibility with international academic and clinical standards. Effectively navigating this challenge requires evidence-informed language policies, commitment to educational equity, and sustained institutional investments.
